# Supplementary material for: The effect of intrapartum prolonged oxygen exposure on fetal metabolic status: secondary analysis from a randomized controlled trial
Source: Front Endocrinol (Lausanne). 2023 Jun 27;14:1204956. doi: 10.3389/fendo.2023.1204956 (PMC10335765; doi:10.3389/fendo.2023.1204956)
Supplement: Supplementary file 3 [file DataSheet_3.zip › Result-X101SC21092977-Z01-J001-B1-42/1.MetQuant-QC/Correlation/cor_pearson_neg.pdf]

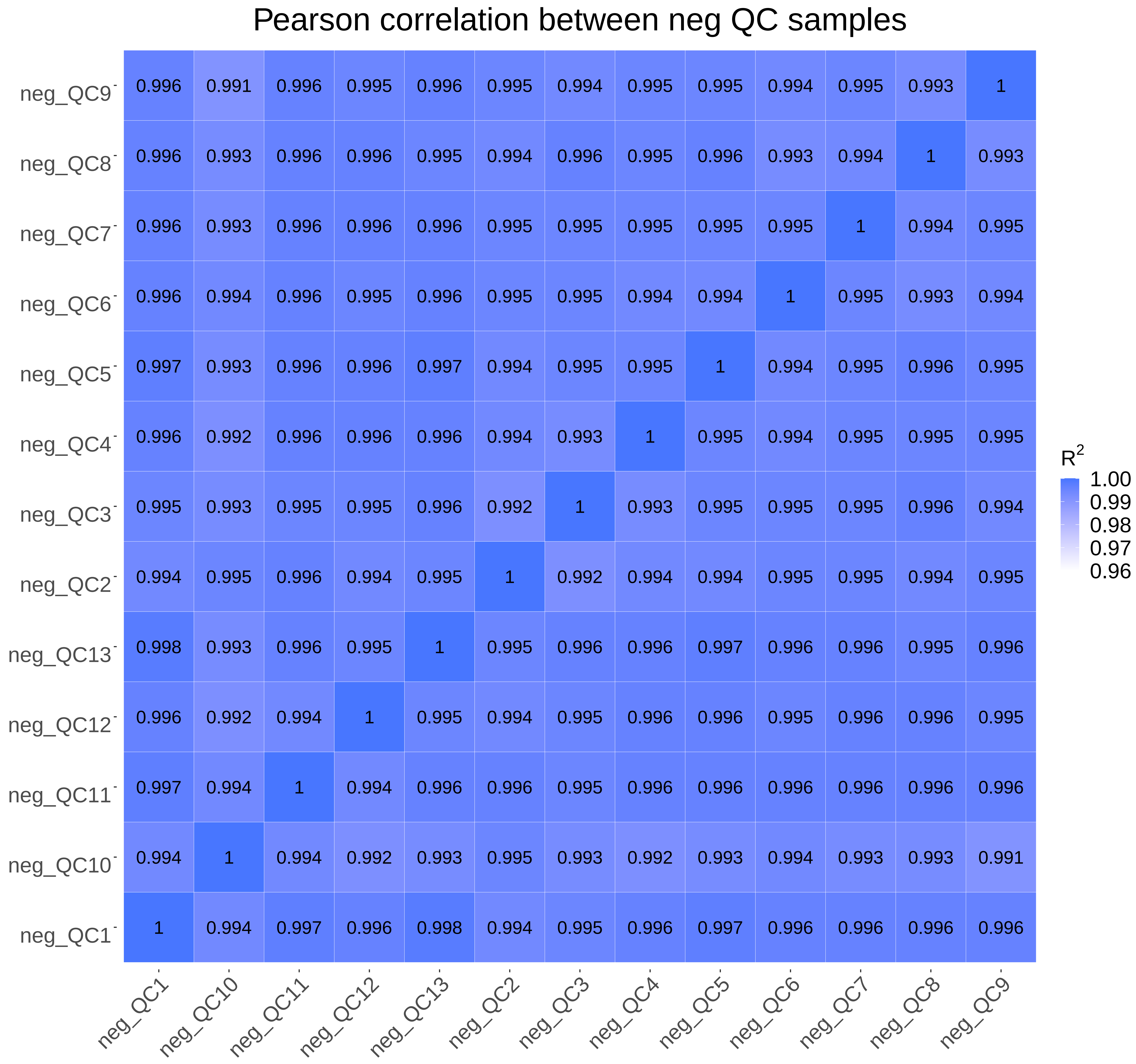
Pearson correlation between neg QC samples
neg_QC9 0.996 0.991 0.996 0.995 0.996 0.995 0.994 0.995 0.995 0.994 0.995 0.993 1
neg_QC8 0.996 0.993 0.996 0.996 0.995 0.994 0.996 0.995 0.996 0.993 0.994 1 0.993
neg_QC7 0.996 0.993 0.996 0.996 0.996 0.995 0.995 0.995 0.995 0.995 1 0.994 0.995
neg_QC6 0.996 0.994 0.996 0.995 0.996 0.995 0.995 0.994 0.994 1 0.995 0.993 0.994
neg_QC5 0.997 0.993 0.996 0.996 0.997 0.994 0.995 0.995 1 0.994 0.995 0.996 0.995
neg_QC4 0.996 0.992 0.996 0.996 0.996 0.994 0.993 1 0.995 0.994 0.995 0.995 0.995
neg_QC3 0.995 0.993 0.995 0.995 0.996 0.992 1 0.993 0.995 0.995 0.995 0.996 0.994
neg_QC2 0.994 0.995 0.996 0.994 0.995 1 0.992 0.994 0.994 0.995 0.995 0.994 0.995
neg_QC13 0.998 0.993 0.996 0.995 1 0.995 0.996 0.996 0.997 0.996 0.996 0.995 0.996
neg_QC12 0.996 0.992 0.994 1 0.995 0.994 0.995 0.996 0.996 0.995 0.996 0.996 0.995
neg_QC11 0.997 0.994 1 0.994 0.996 0.996 0.995 0.996 0.996 0.996 0.996 0.996 0.996
neg_QC10 0.994 1 0.994 0.992 0.993 0.995 0.993 0.992 0.993 0.994 0.993 0.993 0.991
neg_QC1 1 0.994 0.997 0.996 0.998 0.994 0.995 0.996 0.997 0.996 0.996 0.996 0.996
neg_QC1 neg_QC10 neg_QC11 neg_QC12 neg_QC13 neg_QC2 neg_QC3 neg_QC4 neg_QC5 neg_QC6 neg_QC7 neg_QC8 neg_QC9
R2
1.00
0.99
0.98
0.97
0.96
